# *ResynPy*: a software for selecting pairs of complementary inbred lines to resynthesize valuable heterozygous genotypes

**DOI:** 10.1186/s12859-025-06279-x

**Published:** 2025-10-28

**Authors:** Konstantinos G. Alexiou, Iban Eduardo, Pere Arús

**Affiliations:** 1 IRTA, Campus UAB, Edifici CRAG, Cerdanyola del Vallès, Bellaterra, Barcelona 08193 Spain; 2 Centre for Research in Agricultural Genomics (CRAG) CSIC-IRTA-UAB-UB, Campus UAB, Edifici CRAG, Cerdanyola del Vallès, Bellaterra, Barcelona 08193 Spain

**Keywords:** Parental selection, Resynthesis, Python, Parallelization

## Abstract

**Background:**

Using cultivars based on a single, partly heterozygous, genotype is one of the bases of agriculture, frequently used in seed-propagated species such as vegetables and field crops (F1 hybrid varieties), and in most fruit trees and ornamentals (clonal varieties) that reproduce vegetatively. Producing two inbred lines that fully or partly reconstruct a given high-value heterozygous individual is now possible with the Resynthesis method, which uses molecular markers to select pairs of complementary genotypes in the F2 and subsequent selfing generations. The objective of this paper was to develop a software to facilitate the selection of such complementary pairs from a large number of segregating genotyped individuals.

**Results:**

The *ResynPy* tool we developed screens the genotyping data of a segregating population, originating from selfing a top-performing partly heterozygous individual, and detects pairs of individuals that could produce progeny containing individuals nearly identical to the initial elite genotype. Our software selects candidate pairs of individuals in a small timeframe window with a low memory footprint. *ResynPy* tool could be part of a breeding toolkit to accelerate breeding programs in any plant species of economic importance capable of producing self-pollinated progeny.

**Conclusions:**

*ResynPy* is a software developed in Python language and is freely available on GitHub (HYPERLINK “https://github.com/kostasgalexiou/ResynPy/tree/main”). *ResynPy* automates the process of individual selection implemented by the Resynthesis method, in a highly efficient manner through parallelization, allowing to plant breeders for detection of pairs of complementary individuals in a selfing population that could be suitable parents to a plant variety with economically important agronomic traits. With *ResynPy*, breeders are provided with a fast and easy-to-use tool that will aid them to advance their plant breeding programs.

**Supplementary Information:**

The online version contains supplementary material available at 10.1186/s12859-025-06279-x.

## Background

Resynthesis is a new marker-based breeding strategy developed to obtain slightly modified elite partly heterozygous cultivars (F1 or clonal varieties) with new agronomic traits of interest within a window of 1–3 selfing generations [1]. Implementation of this strategy involves generating a large F2 population from the cultivar of interest for the marker-based selection of highly homozygous pairs of individuals with complementary genotype profiles. Selected individuals from the F2, or more advanced selfing generations, also selected with markers to be further fixed and complementary, can be crossed to produce individuals highly similar to the elite cultivar but with new desirable traits conferred by the internal hybrid variability (see a scheme of the selection process in Additional file 1). Resynthesis also facilitates the integration of genes of interest from other sources in one of the complementary lines after one or two marker-assisted backcross generations [2], and subsequent crossing to a complementary pair, producing a cultivar similar to the original with new desirable traits. The software we developed, *ResynPy*, automates the process of individual selection, making the use of Resynthesis with the species of choice accessible to researchers and breeders even with limited bioinformatics skills.

## Implementation

*ResynPy* is intended to help breeders to select the complementary couples of the Resynthesis breeding strategy [1]. In the initial selfing generations (F2-F4), a large number of seedlings in the greenhouse is selected based on their genotypes and then a limited number of them will be transplanted and used for further genetic analysis. The typical size of these F2-F4 populations is extremely high (*N* = 1000–10,000 in each generation), especially in the F2 where it is easier to have larger progenies compared to further selfing generations the size of which usually depends on the reproductive capability of a single individual. For economic reasons, it is advisable to use a low number of high-quality markers (usually SNPs), covering well the chromosomes at distances of 10–15 centimorgans (cM) to assure the detection of most double recombination events on each chromosome. A typical diploid or allo-polyploid crop species has a range of 2n = 10–50 chromosomes with 1–3 recombinations per meiosis and chromosome [3]. A species with an exceptionally high recombination rate (3 recombinations per chromosome) and high chromosome number (2n = 48), would require 384 markers: 16 well-distributed markers per chromosome, one every 10 cM, of 24 chromosomes of ~ 150 cM each, for a reasonable genome coverage. However, such species would need a higher number of progeny to progress in the selection of complementary couples compared to one with lower number of chromosomes or recombination rates. Further saturation with markers can be done in a selected set of couples (20–100, depending on the generation, see Additional file 1) previously chosen with the help of *ResynPy*, and has a straightforward interpretation that does not need automation.

The *ResynPy* software accepts two tabulated files as mandatory input arguments: a genotyping file and a markers file. The genotyping file contains the genotype data of the F2 population represented as “A”, “B”, “H”, “-”, where “A” is the homozygous genotype for one parental allele, “B” for the other parental allele, “H” the heterozygous genotype and “-” represents the missing data. Markers are in columns and individuals in rows. The markers file is formatted in two columns, with the first one containing the chromosome name and the second one the marker name. Marker order in the genotyping and marker files should be identical. Process optimisation was achieved by parallelization of the analysis across multiple processing core (CPUs), resulting into a drastic ~ 10X reduction of processing time compared to running times without parallelization (see Results).

The software starts by keeping only individuals with a heterozygosity rate lower than a certain value, which can be modified by the user. In the test data used (see Results) we chose this value to be 0.5, which would discard approximately half of the individuals of an F2 population. Following heterozygosity filtering, the program performs unidirectional pairwise comparisons for every individual in the population. The number of unidirectional comparisons can be calculated by the formula (n * (n – 1)) / 2, where “n” represents the number of individuals. Each comparison is made per marker, looking at the genotypes of each pair at the locus. A score in the range of 0 to 1 is assigned to each locus depending on the combination of genotypes found between individuals in each pair (“AB”, “AH”, “BH”, “HH”, “AA”, “BB”, “A-”, “B-”, “H-” and “--”). The values given to each combination can be determined by the user, even though we offer a set of default scores to use, tolerant to missing data and heterozygosity. Scores assigned at each marker locus are added together and this sum (the final score) is assigned to each pair of individuals. Final scores that fall below a user-defined threshold of the ideal score, corresponding to the score obtained if we had a pair of completely complementary individuals, are also discarded by the software to speed up the process. Additional filtering is applied at the level of invariable site presence, i.e., pairs of loci with the same allele in both individuals (“AA” or “BB”). This parameter is chosen by the user, with typical values ranging from zero, which would discard all pairs with one or more homozygous markers, to 0.1, which will be more adequate to find complementary pairs when using a relatively small number of individuals.

*ResynPy* also estimates the number of recombinations of each individual, as well as the sum of the recombination events of both haplotypes of the pair analysed. These are only calculated if the genotyping data are phased and can be used as an additional step of filtering, in case of having to select among pairs with the same final score. Those with the lowest number of recombinations are usually the most desirable, as every recombination event generally implies that a larger or smaller homozygous DNA fragment will occur at that region in the hybrid plants, considering that it is highly improbable that both members of a complementary pair have a recombination breakpoint at the same position.

To represent the degree of similarity between the resynthesized genotype obtained by selected pairs of individuals and the original hybrid genotype (the progenitor of the F2 population), we use a range of percentage values. The upper end of the range represents the highest degree of similarity to the hybrid, after removing the invariable markers (“AA” or “BB”). For example, for a genotype composed of 50 markers, where four of them are invariable between the individuals, the highest ratio of similarity will be 92% [(50–4) * 100 / 50 = 92]. The lower end of the ratio is calculated by subtracting, apart from the invariable sites, those where at least one of the individuals is heterozygous. If ten heterozygous markers are present in our previous example, the lower end of the similarity ratio would be 72% [(50–4 – 10) * 100 / 50 = 72].

The pipeline was run on a desktop PC (15,5 GiB memory, Ubuntu 20.04.5 LTS, Intel^®^ Core™ i7-6700 CPU @ 3.40 GHz × 8).

## Results

**Fig. 1:**
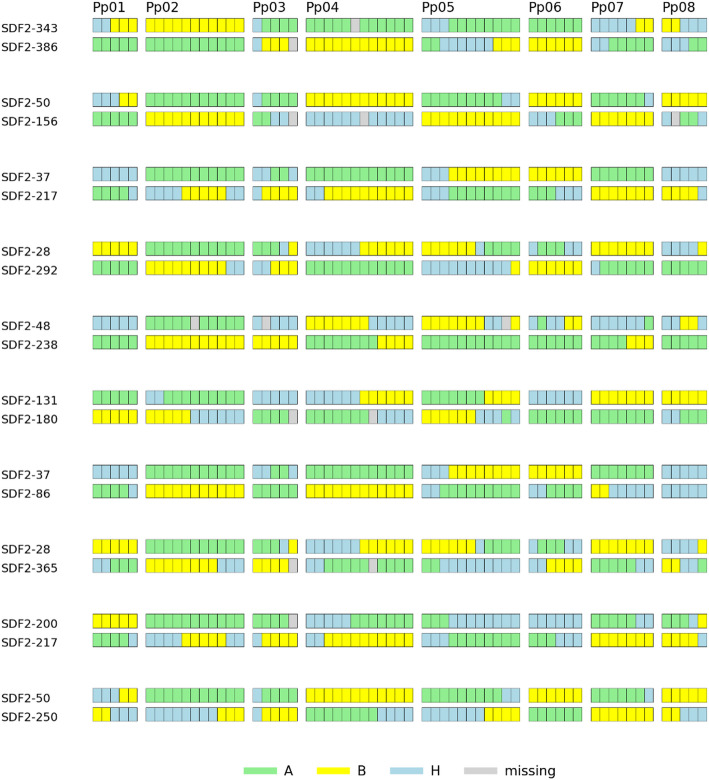
Graphical representation of genotypes for the pairs of individuals with the 10 highest scores in the ‘Sweet Dream’ peach F2 progeny. The left column indicates the genotype name, and the first row corresponds to the eight peach chromosomes (Pp01 to Pp08)

*ResynPy* organizes the information into four groups. The major output is a file containing the accepted pairs of individuals, the final score of the pair, the number of invariable sites, the ratio of heterozygosity present in each individual and the range of similarity to the hybrid genotype. If genotyping data are phased, the output file is supplemented by three additional columns: the number of recombinations in the first individual, the number of recombinations in the second individual and the total number of recombinations (Additional file 3). The second file contains all the discarded individuals or pairs of individuals, annotated with the corresponding filtering parameter (Additional file 4). A third file is a log containing the arguments applied by the user and statistics for the filtering process (Additional file 5). The final figure generated (.png and .pdf formats) is a graphical representation of the genotypic profiles of the individuals in the top ten pairs (Fig. 1).

Our test data consisted of a set of 418 F2 individuals of peach cultivar ‘Sweet Dream’ genotyped with 62 SNP markers with good coverage of the peach genome used by [1]. These markers were phased, and the proportion of missing data was 2%. It took only 7 s to run our *ResynPy* pipeline on the test data. Applied filters of ≤ 50% heterozygosity, ≤ 1% invariant pairs, and with a set of our default scores, led to the detection of 46 pairs of individuals with scores ranging from 49.6 to 55.0 (ideal score: 62), all of them with zero invariable pairs and with 31 pairs (67%) with no recombination events (Additional file 3). Manual selection of the best pairs was implemented by [1], trying to find optimal combinations of low heterozygosity in the parents and high complementarity in the couples. In that paper, four pairs were selected, three of which (SDF2-343|SDF2-386, SDF2-37|SDF2-217 and SDF2-131|SDF2-180) coincided with the three top pairs selected by *ResynPy*. The fourth pair, SDF2-238|SDF2-412, is not found in the *ResynPy* output file, but it was selected by [1] following other criteria, as SDF2-238 is an exceptional plant, homozygous for all markers tested, and the objective was to find the best complementary pair with zero invariable loci for this peach pure line. The selected partner, SDF2-412, has an overall heterozygosity of 0.56, and thus was discarded by the pipeline due to the 0.5 threshold applied to the selected F2 progeny.

Overall, these data indicate a close agreement between the results of [1] and those generated with *ResynPy*, with the difference that to obtain the former was a time-consuming operation, while those of *ResynPy* were produced in a matter of seconds. On the other hand, the number of plants used in the ‘Sweet Dream’ example was relatively low, and the interest of using *ResynPy* increases when using much larger sets (i.e., thousands) of F2 progeny or in species with more chromosomes, more recombination events per chromosome, or both.

*ResynPy* pipeline is species-independent and can support higher numbers of datapoints than those used in the public dataset above. To demonstrate its upscaling capability, we used synthetic genotyping data obtained by software *pedigreeSim*, which generates simulated genetic marker data of individuals belonging to a specific pedigree [4]. Briefly, we simulated genotyping data for 500 markers in an F2 population of 10,000 individuals for a species composed of 10 chromosomes (Additional file 6). Each chromosome was assigned a length of 100 cM and was genotyped with 50 markers, distributed in an equidistant manner across its length. To mimic real conditions, we also generated a modified dataset that included 5% of missing data. Running time for our pipeline without parallelization, with ≤ 50% heterozygosity, ≤ 10% of invariable loci and ≥ 60% of the ideal score on genotyping data for all the 10,000 individuals, was 101 ± 6.0 and 98 ± 6.0 min, on average, without and with 5% missing data, respectively. With the implementation of the parallelization protocol the corresponding times have fallen to 11 ± 2.1 and 12 ± 2.7 min, respectively, showing a drastic reduction in pipeline’s processing time (Additional file 7). The total amount of pairs that was compared amounted to more than 12 million, applying the 50% heterozygosity filtering. To evaluate the results on different parameters (final score, % heterozygosis, % of invariants) under three different score scenarios (Table 1), we used the two types of simulated data mentioned above. We calculated the average values for the set of 100 highest ranking pairs (Table 2) and we observed that, as expected, the data from Set 1 (intolerant to missing data) and Set 3 (tolerant to missing data and heterozygosis) were the same for the complete set of data and different for the data with 5% missing values, with Set3 selecting pairs with a higher final score. The Set 2 (with low tolerance to heterozygosis) presented a slight decrease in heterozygosis in both datasets, although at a high cost in final score and % of invariants.

**Table 1:** Three different examples of sets of scores that were assigned to each genotype pair during the comparison of two individuals

Genotype pair	A/A	B/B	H/H	–/–	H/–	A/–	B/–	A/H	B/H	A/B
Set1	0	0	0.5	0	0.5	0	0	0.75	0.75	1.0
Set2	0	0	0.25	0	0.25	0.5	0.5	0.5	0.5	1.0
Set3	0	0	0.5	0.5	0.625	0.75	0.75	0.75	0.75	1.0

Set1 is intolerant to missing data, Set2 provides high preference to homozygous A/B combinations and Set3 is more tolerant to missing values and to heterozygous genotypes

**Table 2:** Average final score (FScore), percentage heterozygosity (%Het) and percentage invariant genotypes (%Inv) of the best 100 complementary pairs (those with the highest score) selected with *ResynPy*, with %Het ≤ 50 and %Inv ≤ 10, in a simulated F2 progeny of *N* = 10,000 (x = 10, 50 markers and 100 cM per chromosome)

	All datapoints			5% missing data		
	F score	% Het	% Inv	F score	% Het	% Inv
Set 1	419.13	29.97	1.17	389.19	31.27	1.04
Set 2	358.53	28.39	2.34	344.72	28.25	2.29
Set 3	419.13	29.97	1.17	413.02	29.94	1.05

Set 1 scores are for a scenario intolerant to missing data, Set 2, intolerant to heterozygosis and Set 3 with some tolerance to both, heterozygosis and missing data

## Conclusions

We developed *ResynPy*, a plant species-independent and time-efficient pipeline that automates the process of selection of pairs of complementary individuals with the Resynthesis method [1]. This pipeline can be used without prior knowledge of command-line interfaces. *ResynPy* allows for rapid selection of plants, without the breeder having to resort to laborious and potentially error-prone manual visualization and selection of the desired individuals. It offers ample flexibility to the user, permitting the control of different population parameters (individual heterozygosity, invariable sites, and % final score over the ideal score) as well as the scoring scale, with the latter making it possible to modify the weight that each genotype pair combination at each locus has on the final score. Marker data from the F2 of a partly heterozygous individual is usually unphased, as the genotypes of the parents and grandparents are rarely known. This information is optional for running *ResynPy*, except for the recombination event estimation. Considering that the available genotyping data covers well the mapping distance of the chromosomes of the species studied, at distances not exceeding 10–25 cM, phasing is easily done by visual examination of the data or by constructing a linkage map with mapping software that allows unphased data. An issue that the user should consider is the proportion of missing data in the population, since their inherent uncertainty can jeopardize the selection of the best pairs from the population. We recommend that, if missing data is an issue, there should be a previous imputation step applied, using one of the different software available, such as Beagle [5], fastPHASE [6] or IMPUTE2 [7].

## Supplementary Information

Below is the link to the electronic supplementary material.

Additional file 1.

Additional file 2.

Additional file 3.

Additional file 4.

Additional file 5.

Additional file 6.

Additional file 7.

## Data Availability

Datasets generated and/or analysed during the current study are available in the *ResynPy* repository, https://github.com/kostasgalexiou/ResynPy. Real genotyping data produced by [1] and used for testing our pipeline is provided in Additional file 2.
