# Supplementary material for: ResynPy: a software for selecting pairs of complementary inbred lines to resynthesize valuable heterozygous genotypes
Source: BMC Bioinformatics. 2025 Oct 28;26:265. doi: 10.1186/s12859-025-06279-x (PMC12560546; doi:10.1186/s12859-025-06279-x)
Supplement: Supplementary file 1 — Additional file 1. [file 12859_2025_6279_MOESM1_ESM.pdf]

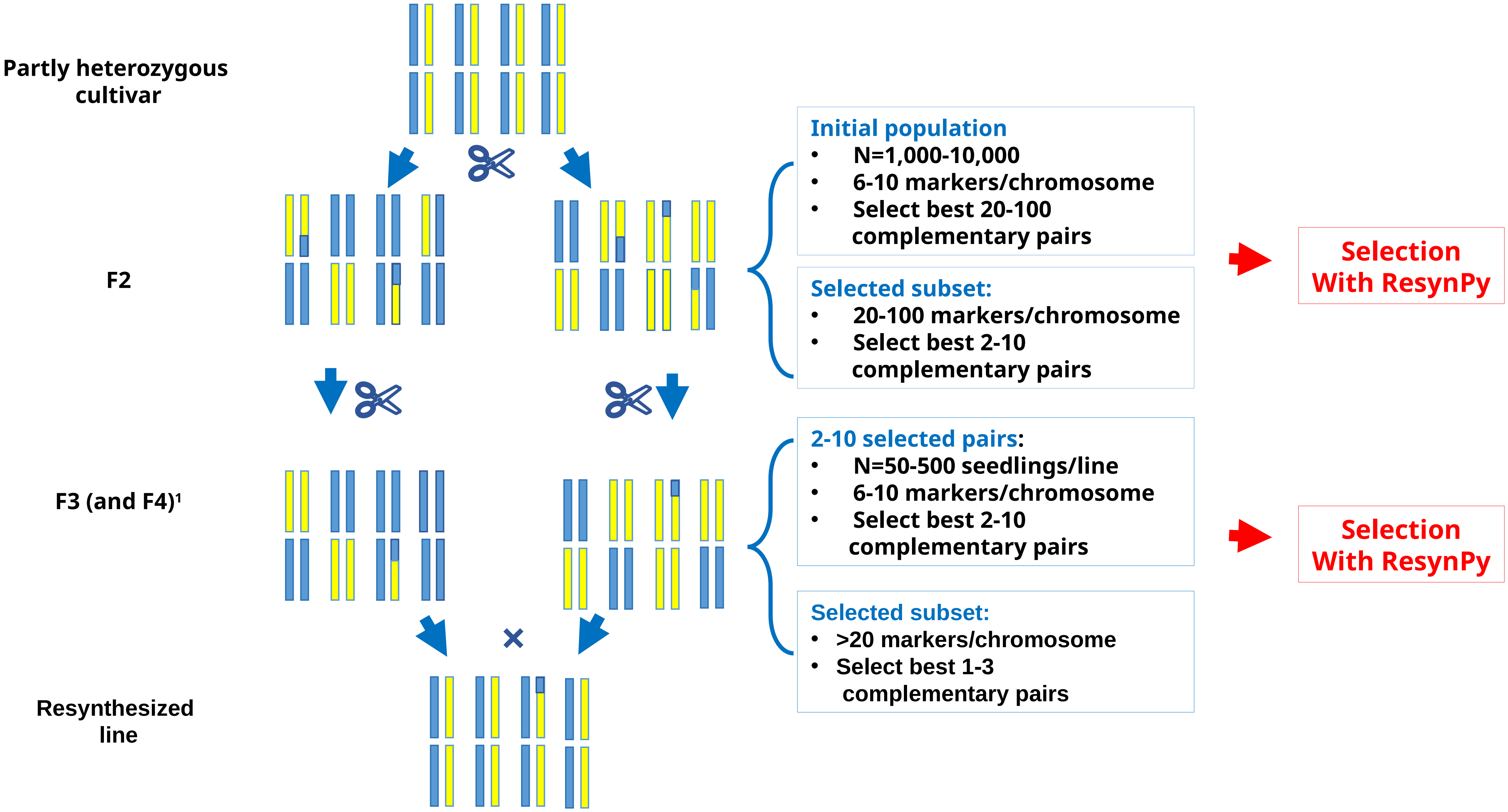

Supplementary Figure 1: Basic scheme of the resynthesis process with appoximate typical numbers of individuals and markers used at each phase, including the steps of the selection process where *ResynPy* may be used.

[1]The F4 generation may not be necessary depending on the results of the previous generations, which may depend on the numbers of plants used and the rate of recombination and number of chromosomes of the species used.
